# PoolHap: Inferring Haplotype Frequencies from Pooled Samples by Next Generation Sequencing

**DOI:** 10.1371/journal.pone.0015292

**Published:** 2011-01-05

**Authors:** Quan Long, Daniel C. Jeffares, Qingrun Zhang, Kai Ye, Viktoria Nizhynska, Zemin Ning, Chris Tyler-Smith, Magnus Nordborg

**Affiliations:** 1 Gregor Mendel Institute, Vienna, Austria; 2 The Wellcome Trust Sanger Institute, Cambridge, United Kingdom; 3 Research Department of Genetics, Evolution and Environment, University College London, London, United Kingdom; 4 Beijing Institute of Genomics, Chinese Academy of Sciences, Beijing, People's Republic of China; 5 Departments of Molecular Epidemiology, Medical Statistics and Bioinformatics, Leiden University Medical Center, Leiden, The Netherlands; 6 Molecular and Computational Biology, University of Southern California, Los Angeles, California, United States of America; Aarhus University, Denmark

## Abstract

With the advance of next-generation sequencing (NGS) technologies, increasingly ambitious applications are becoming feasible. A particularly powerful one is the sequencing of polymorphic, pooled samples. The pool can be naturally occurring, as in the case of multiple pathogen strains in a blood sample, multiple types of cells in a cancerous tissue sample, or multiple isoforms of mRNA in a cell. In these cases, it's difficult or impossible to partition the subtypes experimentally before sequencing, and those subtype frequencies must hence be inferred. In addition, investigators may occasionally want to artificially pool the sample of a large number of individuals for reasons of cost-efficiency, e.g., when carrying out genetic mapping using bulked segregant analysis. Here we describe PoolHap, a computational tool for inferring haplotype frequencies from pooled samples when haplotypes are known. The key insight into why PoolHap works is that the large number of SNPs that come with genome-wide coverage can compensate for the uneven coverage across the genome. The performance of PoolHap is illustrated and discussed using simulated and real data. We show that PoolHap is able to accurately estimate the proportions of haplotypes with less than 2% error for 34-strain mixtures with 2X total coverage *Arabidopsis thaliana* whole genome polymorphism data. This method should facilitate greater biological insight into heterogeneous samples that are difficult or impossible to isolate experimentally. Software and users manual are freely available at http://arabidopsis.gmi.oeaw.ac.at/quan/poolhap/.

## Introduction

There are many situations in which we would like to determine the haplotype composition of a polymorphic sample or population. However, while rapidly decreasing sequencing costs are making it feasible to accomplish this by simply sequencing large numbers of individual samples from the population (perhaps using bar-coding), it is often impossible or very costly to obtain individual samples, because it would involving labor-intensive cloning and culturing. Currently, to analyze different pathogen strains, scientists usually culture samples to isolate them and sequence them separately[1], [2], [3]. However, determining the relative frequencies of strains within hosts this way would be prohibitively expensive. Analogously, in cancer studies, techniques have been developed for identifying and extracting oncocyte from tissue before doing further analysis[4], [5], [6], but determining the proportions of different cell lineages in tissue this way is not practicable. In the field of mRNA expression studies, many efforts have gone into experimental methods for isolating alternatively spliced isoforms[7], [8], but these are again not suitable for high-throughput analysis. Besides such general applications, there are many specific problems involving pooled haplotype analysis. A recent example is mitochondrial heteroplasmy, which is believed to impact aging[9]. If one wants to use sequencing to do further investigation on the haplotype level (instead of single marker level), a haplotype analysis tool is needed.

In addition to naturally pooled samples, it is sometimes sensible to pool samples artificially simply to reduce cost. For example, it is going to be common to pool samples with extreme phenotypes to do association mapping[10], and it is also possible to envision monitoring haplotype frequency changes in cohorts by sequencing. As long as it is possible to infer haplotype frequencies, low-coverage sequencing (the total coverage could be the same or even smaller than number of haplotypes) may well be more cost-effective than sequencing a large number of individuals.

To facilitate those applications, we provide PoolHap, a computational tool for inferring haplotype frequencies from pooled samples when haplotypes are known. The PoolHap pipeline assumes that the investigators have sequenced a pool of samples and have run a mapping tool (e.g. BWA[11]) to map the short reads to a reference genome to call SNPs from the consensus sequence. For each bi-allelic heterozygote variation called, PoolHap calculates the ratio between numbers of reads supporting the two alleles and the total coverage as the evaluated allele frequencies. Then PoolHap infers the haplotype frequencies.

The key insight into why PoolHap works is that the very large number of SNPs that come with genome-wide coverage can compensate for the uneven coverage across the genome. We use a regression model. Assume we have *h* potential known haplotypes and many SNPs in which we can choose *n* most *informative* bi-allelic SNPs (See **Methods** for how we define and find *informative* SNPs). We model the allele frequencies vector (observed in the assembly) as a dependent variable *Y*; SNP alleles in haplotypes as independent variables *X_1_*, …, *X_h_*, where each *X_i_* is a *n*-vector composed of 0 and 1 regarding to the alleles in the corresponding *i*th haplotypes; haplotype frequencies as coefficients *b_1_*, …, *b_h_*. Then solving the regression *Y = b_1_X_1_+*…*+b_h_X_h_* yields the estimated haplotype frequencies (see **Methods** for precise formalization and methodology). The advantage of this model is that (1) the estimation of frequencies does not suffer from co-linearity between *X_i_*s and (2) it is robust to coverage bias between regions (see **Methods** for detailed arguments). In what follows, we will examine the performance of PoolHap under various conditions using both simulated and real data, and discuss its advantages as well as limitations.

## Results

### Inferring the frequencies of known haplotypes in a pooled sample of simulated *Arabidopsis thaliana* data

To examine how the performance of PoolHap depends on SNP number and sequencing coverage when haplotypes are known, we use simulations based on real sequencing data from *A. thaliana*, generated as part of the 1,001 genomes project (http://www.1001genomes.org). We have sequenced the whole genome of over 50 Swedish strains. There are around 20 million 76 bp paired-end reads for each strain, yielding around 20x coverage. We map all the reads to col-0 reference to call the SNPs by BWA and SAMtools[12]. The number of SNPs of these strains ranges from 183,883 to 523,756. Because these strains are inbred lines, most of the SNPs are homozygous.

**Figure 1:**
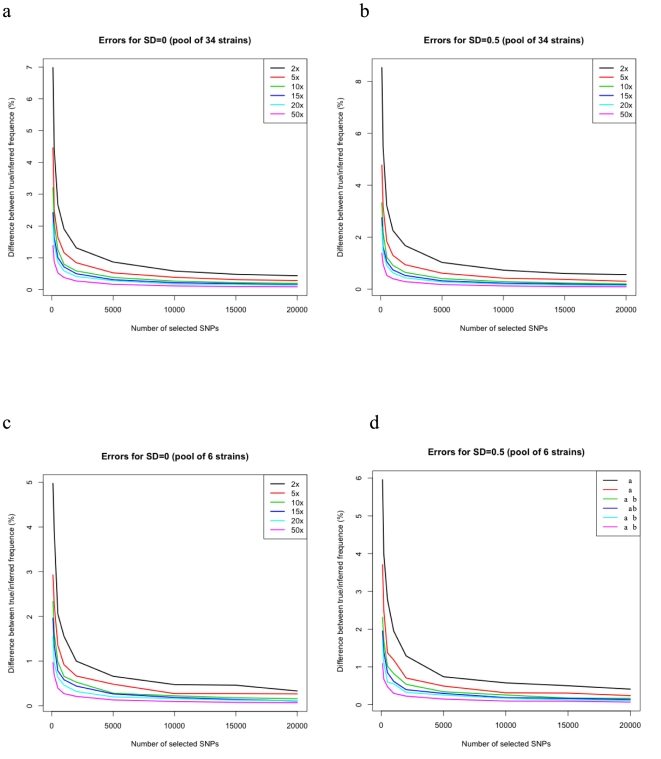
Error estimation of PoolHap applied on simulated whole-genome sequencing based on real SNPs of *A. thaliana.* The *y*-axis is the average % difference (real – predicted, absolute value), the *x*-axis is the number of selected SNPs; different curve in the same panel stands for different total coverage of the pool. Panel (a) is the pool of 34 strains with coverage standard deviation SD = 0, and (b) is the pool of 34 strains with coverage standard deviation SD = 0.5. Panel (c) is the pool of 6 strains with coverage standard deviation SD = 0, and (d) is the pool of 6 strains with coverage standard deviation SD = 0.5.

Based on the above SNPs identified in read data, pools of 6 strains and 34 strains were simulated. For each simulation, we select all SNPs that show up in 40%∼60% of the strains that have SAM quality score[12] 255 (which is the highest score) and are bi-allelic in all strains as the candidate SNPs. We specify the number of SNPs to be used, and select these as the most informative ones using PoolHap's SNP-selection function. Finally, we generate random samples to achieve a pre-determined mean and standard deviation (SD) of coverage. We use the average absolute value of differences between true and inferred frequencies as a matrix of error. For each combination of mean of coverage, SD of coverage, and number of SNPs, we replicate 30 random frequencies and take the average of the errors as final error. The results of 34 strains are depicted in **Figure 1**(**a**) and (**b**), and the 6 strains are depicted in **Figure 1**(**c**)and (**d**).

We find that even with as little as 2x coverage (standard deviation 0.5), and 1,000 selected SNPs (from 189k SNPs) PoolHap is able to estimate the haplotype frequencies to within 2% of their true value (**Figure 1**(**b**)). Results are only slightly better when coverage standard deviation is reduced to zero (**Figure 1**(**a**)), indicating that PoolHap algorithm is free of stochastic coverage which frequently occur with NGS. Errors reduce to less than 1% with increasing coverage of selected SNPs. These observations confirm the intuition that PoolHap can successfully take advantage of large number of SNPs to correctly infer haplotype frequencies with low and uneven coverage.

### Simulated *A. thaliana* gene expression data

PoolHap can also be applied to RNA-Seq data to infer the relative abundances of different transcripts of the same gene. In theory, the situation is the same as other haplotype-known problems. We can just simply encode the exons as if they are SNPs: if the exon is presence in one isoform, then we encode it as 1, otherwise 0. However, there are some differences in this particular application: (1) some isoforms share the same exon but have different 5′ or 3′ termini; (2) the coverage of the first and last exons will often be artifactually lower. To facilitate this application, we developed a sub-function special for RNA-Seq (Method). We simulate *A. thaliana* RNA-Seq data based on the gene models downloaded from TAIR website (www.arabidopsis.org) with random frequencies. Using the same reads simulation procedure and the same error measurement to the former simulation, we assess the performance of PoolHap on all genes of *A. thaliana* with more than one isoform.

**Figure 2:**
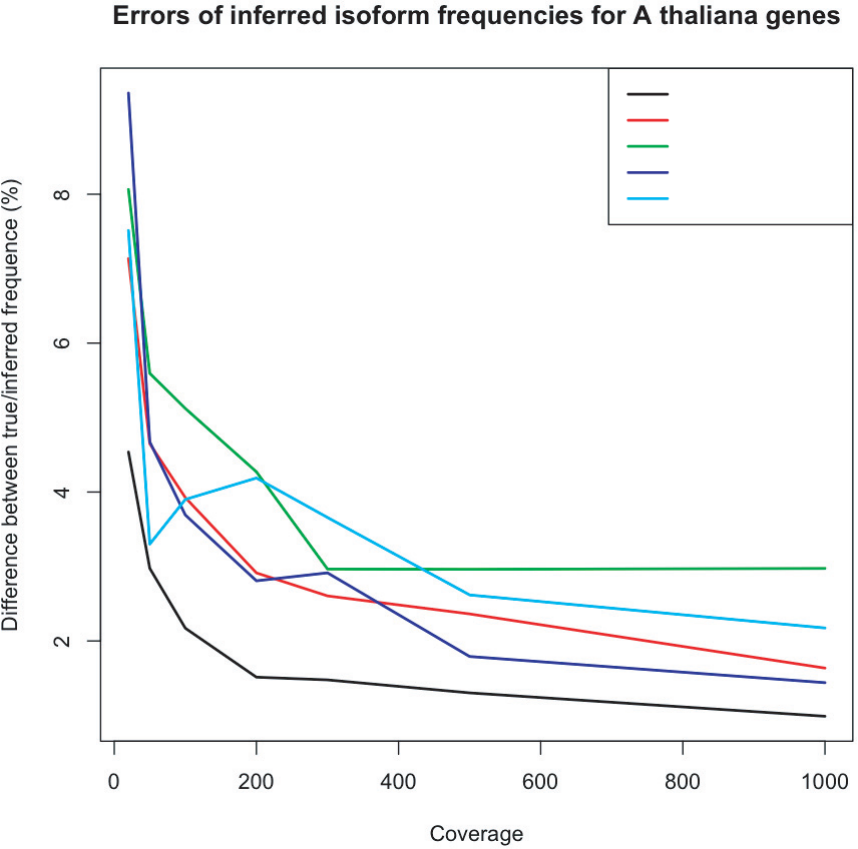
Error estimation of PoolHap applied on simulated RNA-Seq data based on real gene models of *A. thaliana.* The *x*-axis is the total coverage of the pool, the *y*-axis is the average % difference (real – predicted, absolute value). Different curves stand for genes with different number of isoforms, ranging from 2 to 6.

We find that with 50x coverage we can infer the frequencies of three-isoform genes with less than 5% error. Precision is marginally less for more complex genes, and increases with increased coverage. In some genes, because the fact that different isoforms show very small differences or even the same after our processing to deal with problems (1) and (2), the regression solver cannot distinguish different independent variables (i.e., meets singular matrix) therefore fails to find the solution. We treat those cases as “trivial gene model” and omit them in the performance evaluation. The results for the remaining genes are depicted in the **Figure 2**.

As one can see, the performance is not as good as the simulation on whole genome data. The reason is, due to the limited difference between isoforms in some genes, the ability of distinguish them is also limited.

So far we have shown the results on simulated data, which is a simplified vision of real applications. However, a few factors absence in the simulation, e.g., copy number variations, library duplications, sequencing/mapping errors, may be important in real applications. In the following we test PoolHap with real NGS reads.

### Application to a pooled sample of known *A. thaliana* haplotypes

We apply PoolHap to the mixture of a subset of real data of the reads described before. We randomly select a subset of reads from 6 (or 16) strains with predefined proportions, map these reads to the reference genome by BWA, call SNPs at the known polymorphism sites, and then use the PoolHap method to infer the proportions of strains.

The total coverage of this dataset is 20x, which is currently the typical coverage of one Illumina lane for *A. thaliana*. The results are presented in **Table 1** **and** **2** **.** We find that with 6 strains, we correctly infer the proportion of each strain with an average of 1.6% difference between the actually proportion and our predicted proportion. With 16 samples, results are similar, the average difference being 0.6%. The use of mixed read data sets is more difficult than simulations, probably due to library duplications, genome structural rearrangements, sequencing errors, and mapping errors, etc. For comparison purpose, we also list the results inferred from simulation in the same condition to show how the performance decreases slightly with real data.

**Table 1:** Inferred frequencies with pooled Illumina reads of six *A. thaliana* strains and the corresponding results from simulations.

Strain ID	Real Frequency	Inferred frequency	SD of inferred frequency	Inferred freq. in Simulation. (SD = .5)
Lom1_1	1.80%	1.90%	+/−0.41%	1.50%
Ull2_5	4.60%	4.70%	+/−0.41%	4.80%
Kavlinge_1	14.70%	13.20%	+/−0.42%	14.50%
Sr_5	18.60%	20.80%	+/−0.41%	18.30%
Vastervik	21.50%	24.30%	+/−0.40%	21.60%
Sanna_2	38.80%	35.40%	+/−0.40%	38.90%

Coverage  = 20x. Selected SNP number  = 10,000.

**Table 2:** Inferred frequencies with pooled Illumina reads of sixteen *A. thaliana* strains and the corresponding results from simulations.

Strain ID	Real frequency	Inferred frequency	SD of inferred frequency	Inferred freq. in Simulation (SD = .5)
Nyl_2	0.50%	1.30%	+/−0.33%	0.70%
Lis_2	1.30%	2.70%	+/−0.29%	1.30%
Fab_4	3.20%	3.90%	+/−0.31%	3.40%
Omo2_1	4.10%	3.50%	+/−0.30%	4.10%
Kni_1	4.20%	3.90%	+/−0.32%	4.10%
Eden_1	4.80%	4.30%	+/−0.32%	4.60%
Eden_2	4.80%	4.50%	+/−0.31%	4.70%
Eds_1	5.20%	4.30%	+/−0.32%	5.00%
Rev_1	6.00%	6.70%	+/−0.31%	6.00%
Or_1	7.90%	8.00%	+/−0.29%	8.10%
Spr1_2	8.70%	8.90%	+/−0.30%	8.80%
Bil_7	9.20%	8.20%	+/−0.30%	9.10%
Lov_5	9.50%	10.20%	+/−0.31%	9.50%
Tottarp_2	9.50%	8.80%	+/−0.30%	9.60%
Dra3_1	10.10%	9.90%	+/−0.30%	10.00%
San_2	10.80%	11.00%	+/−0.31%	10.70%

Coverage  = 20x. Selected SNP number  = 10,000.

## Discussion

As one can see from both the simulation and real data analysis, the algorithm works well for whole genome *A. thaliana* data. This is partially because of the fact that we have plenty of SNPs to be selected (which is the nature of whole genome resequencing). In case one does not have that many SNPs, the SNP-selection algorithm will have to be modified to select more individual-specific SNPs. This could also improve the performance on rare haplotypes, which is not well handled in the current version of PoolHap. We hope to improve this in future versions of PoolHap.

It should be noted that in the regression equation of haplotype known part of PoolHap, all the regression coefficients must be positive (or zero) and their sum has to be 1. In our implementation, we have not taken advantage of those constraints, partly because we have not found a good way to incorporate them, but also because they provide a means of checking the results. If the results are correct, i.e., close to the real frequencies, they must approximately satisfy the above constraints. If not, it means that there is systematic bias in the coverage, indicating that the results in this run are not reliable (due to copy number variations, sequencing error, or library duplications, etc). If we force the regression to be solved with respect to those constrains, we lose the possibility of this kind of valuable control.

The current version of PoolHap is ready for haplotype-known applications. We are also developing the extensions for haplotype-unknown functions. There are many scenarios where this may be applied, for example, when pathogen DNA has been extracted from a group of patients That may all have infections from parasites of mixed genotypes. The haplotypes in one sample may share some genetic information with the ones in the others. In this case, we can make use of the LD in the population and try to infer the haplotype frequencies by iteratively sampling from the global haplotype distributions like PHASE[13] does.

If one considers short indels as common variations like SNPs, PoolHap is ready to incorporate them into analysis. However, due to the premature status of indel calling in NGS platforms[14], we have not applied it in our real data analysis. We believe this will be feasible in the near future.

## Methods

### PoolHap main algorithm

Assume we have *h* potential haplotypes and many SNPs in which we can choose *n* most *informative* bi-allelic SNPs (See next subsection on how we define and find *informative* SNPs). We model the SNP allele frequencies (observed in the assembly) as a dependent variable *Y*; SNP alleles in haplotypes as independent variables *X_1_*, …, *X_h_*, where each *X_i_* is a *n*-vector composed of 0 and 1 regarding to the alleles in the corresponding *i*th haplotypes; haplotype frequencies as coefficients *b_1_*, …, *b_h_*. Then solving the regression *Y = b_1_X_1_+*…*+b_h_X_h_* yields the estimated haplotype frequencies. Following are more details.

Let us say we have *n* SNPs chosen from the mixed assembly. We model the allele frequency as a dependent variable, and consider the actual frequencies vector (observed in the assembly) as a sample of *n* realizations of this variable. 
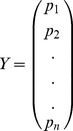
(1)


At the same time, we model each haplotype as independent variable *X_1_*, …, *X_h_*, where each *X_i_* has a realization of an *n*-vector composed of 0 and 1 regarding its allele in at corresponding SNPs.
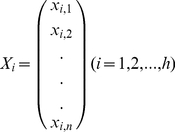
(2)


We model the relationship between the observed assembly and the haplotypes as a random equation:
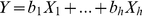
(3)


where coefficients *b_1_*, …, *b_m_* are haplotype frequencies. Then solving the regression *Y = b_1_X_1_+*…*+b_m_X_m_* yields the estimate of haplotype frequencies. If we denote the estimated frequencies as 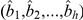, then the error can be specified as 
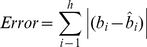
(4)


Here we assume the coverage of different SNPs is independently sampled from the pool, which is approximately true. The exceptions could be (1) there is a duplication/deletion region carrying multiple SNPs. (2) The SNP density is high so that one short read covers multiple SNPs. Nevertheless, this assumption is not crucial in solving the regression.

The first advantage of this model is that it does not suffer from co-linearity between *X_i_*s which frequently happened in regression analysis: it usually will cause an estimate of coefficients of correlated independent variables with very big absolute values and reverse sign as long as their sum is the same. The good point here is that since we model one allele as 0 and the other 1 so that one of the alleles totally does not contribute to the sum. Therefore, only the coefficients on the 1′s take effects in the regression. For example, haplotypes (0,0,0,1) and (1,1,1,0) are strongly correlated but changing the coefficient of (0,0,0,1) cannot compensate for the coefficient change of (1,1,1,0).

Another point is that we model the relationship between haplotypes and mixed assembly and treat SNPs as samples. Thus the biased coverage at a particular SNP or region can be regarded as sampling variance. As long as we have large a number of informative SNPs, i.e., large sample size, we get a good estimate. In this sense, it is robust to uneven coverage of NGS between haplotypes on particular SNPs, as long as the coverage are randomly distributed. For the same reason, it does not matter if we have very low coverage as long as there are sufficient SNPs. However, in case the coverage is systematically biased, which may happen in practice, this model will be biased. An example we observed: there is one strain by which the library construction is biased towards to some regions. When including this strain, the inference of this strain is incorrect and the rest are influenced.

An interesting topic we have not discussed so far is how do we code the alleles. Should we code major alleles are 1 and minors are 0? Or should we code minor alleles are 1? The answer is neither of them is correct. We should just randomly choose an allele to be 1. The reason is that the “major” alleles have to be calculated from the mixed assembly that is not reliable. Consider the following extreme example: there is a SNP with type A/T, and the real allele frequencies of the mixed haplotypes should be 0.5 v.s. 0.5. It is nature that the observed frequency is not exactly the same to 0.5. Then if one fix the coding of major allele as 1, then we find that in all cases, regardless whether the observed frequencies of A is 0.4 or 0.6, we always select an allele with observed frequency bigger than 0.5. Similar situation applies for non-extreme cases as well.

### SNP selection algorithm

As a regression based method, the PoolHap algorithm favourites the configuration in which the correlations between independent variables are small. However, as stated in the above subsection, the special configuration here guarantees that it does not matter if two haplotypes are correlated as long as they have many different homologous alleles. So here the optimal configuration will be reached when the differences between all pairs of haplotypes are as large as possible. More precisely, one should choose the SNPs so that the proportion of different alleles in any two haplotypes is large. Therefore we define the *informative* SNPs as the set of SNPs that maximizes the proportion of different SNPs. In this subsection, we will give general derivation on the average pair-wise differences as a theoretical upper bound and propose our criteria and algorithm with respect to the smallest pair-wise differences afterwards.

From the derivation below, we know that the mean difference of all pairs of haplotypes is fixed when the population allele frequencies are fixed. (Please notice that we denote “allele frequency” as the allele frequencies observed in the assembly in the above subsection. That is, the number of reads supports the alleles divides the number of total reads covering this location. But here, by “allele frequency”, we are referring the number of haplotypes with this allele divide the total number of haplotypes.)

Let us say we have *h* haplotypes and *n* SNPs with alleles frequencies (*f_1_*, …,*f_n_*). We use *h_i_* to denote the *i*th haplotype and *h_i_*_,*m*_ to denote the *m*th SNP at *i*th haplotype. Then the mean difference proportion between pair of haplotypes is
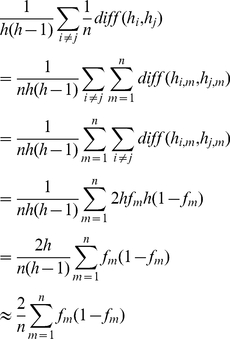
(5)


Therefore we know that the mean of proportion of different SNPs between haplotypes are decided by the population allele frequencies regardless of how the alleles are distributed to the different haplotypes.

The maxima of the above expression can be derived as:
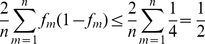
(6)


where the equality holds when, for all *m*, *f_m_* = 0.5.

The above equation gives the upper bound of the performance of SNP selection. However, selecting SNPs with MAF = 0.5 does not necessarily give the best configuration. The reason is that, given the maximal mean difference in the population, there might be a pair of haplotypes with very little difference, therefore the problem similar to co-linearity will happen. So we use the criteria that the smallest proportion of difference among any pairs of haplotypes has to be maximized. To maximize the above measurement, we use the following simple greedy algorithm:

Initially, we randomly choose *n* SNPs as an initial selection, *S_0_*. Then, in the iterative process, from each current selection *S_i_* we identify the pair of haplotypes that reaches the smallest proportion of difference, and change one SNP so that this smallest proportion is increased. If this selection results in a new lower difference between another two haplotypes, we ignore this attempt; otherwise we reach another new selection *S_i+1_*. This process is iterated until either we reach a satisfactory smallest proportion (which is 45% of the SNPs by default, or specified by users) or the number of iterations reaches a pre-specified threshold. We replicate this process 20 times to avoid local maxima.

In theory, this greedy algorithm does not guarantee the global performance. However, in practise, we found that the smallest proportion approaches the upper bound given in formula (5) within around 10,000 iterations when we wanted to find 20,000 SNPs from whole genome *A. thaliana* data. When the candidate SNPs have the right MAF to maximize the upper bound to (6), this algorithm can also approach that.

In practice, if the number of SNPs needed is relatively small compared with the whole data, PoolHap SNP selection program will halt easily. Otherwise, it will try to find the SNPs with lower quality. The users can specify the number of SNPs needed in the analysis.

Besides the above procedure of selecting informative SNPs, we also suggest that users select SNPs to avoid mapping errors, SNP calling errors, as well as structural rearrangements. The current mappers for NGS (e.g., BWA[11]) usually gives SNP quality and coverage of the SNPs. We suggest the users to select SNPs with high SNP qualities and mediate coverage. But we do not provide this function as part of PoolHap pipeline due to many existing mappers and SNP calling methodologies and file formats.

### Sub-function for RNA-Seq data

The problem of detecting frequencies of multiple mRNA isoforms from RNA-Seq data is simply modified to be dealt with by the PoolHap method. As stated in the RNA-Seq simulation section, there are two small problems in making use of the PoolHap algorithm directly on RNA-Seq data: (1) some isoforms share the same exon but not exactly the same coordinates; (2) the coverage of the first and last exons will be lower than the one it should be according to the actual frequency because of the length of the reads. Therefore, in addition to the main algorithm, we developed a sub-function to process RNA-Seq data.

For problem (1), analogous to the approach adopted in by Jiang and Wong[15], we treat the difference between different isoforms on the same exon as another exon. For example, if isoform A and B share the same exon from rough location at chromosome 2 and coordinates 100 to 200+. But, exactly, the exon at A is from 100 to 200, and B is from 100 to 210. Then we extract 200 to 210 as another exon so that A and B share a new exon from 100 to 200, but B has another “exon” from 200 to 210 whereas A does not.

For problem (2), we check the length of first and last exon (the pseudo-exon generated in the last step also counted). If they are not significantly longer than the read length, then we remove them before entering the regression. We use a cut-off that the exon length must be larger than 5 times of the reads. In practice, users can tune this parameter.

Please note that the data this sub-function is working on is the isoforms generated by different gene models. In case the user wanted to get relative abundance of paternal and maternal transcripts by looking at heterozygotes SNPs in the mRNA, s/he will need to use the standard PoolHap, treating paternal/maternal transcripts as known haplotypes.
